# Promising Materials in the Fight against Healthcare-Associated Infections: Antibacterial Properties of Chitosan-Polyhedral Oligomeric Silsesquioxanes Hybrid Hydrogels

**DOI:** 10.3390/jfb14080428

**Published:** 2023-08-17

**Authors:** Antonio Laganà, Alessio Facciolà, Daniela Iannazzo, Consuelo Celesti, Evelina Polimeni, Carmelo Biondo, Angela Di Pietro, Giuseppa Visalli

**Affiliations:** 1 Department of Biomedical and Dental Sciences and Morphofunctional Imaging, University of Messina, 98125 Messina, Italy; antonio.lagana1@studenti.unime.it (A.L.); alessio.facciola@unime.it (A.F.); angela.dipietro@unime.it (A.D.P.); 2 Istituto Clinico Polispecialistico C.O.T., Cure Ortopediche Traumatologiche s.p.a., 98124 Messina, Italy; 3 Department of Electronic Engineering, Industrial Chemistry and Engineering, University of Messina, 98166 Messina, Italy; diannazzo@unime.it (D.I.); consuelo.celesti@unime.it (C.C.); 4 Department of Human Pathology, University of Messina, 98125 Messina, Italy; evipolimeni@gmail.com (E.P.); carmelo.biondo@unime.it (C.B.)

**Keywords:** healthcare-associated infections, chitosan, chitosan-POSS, hydrogel, antibacterial activity

## Abstract

New technologies and materials could help in this fight against healthcare-associated infections. As the majority of these infections are caused by antibiotic-resistant bacteria, the development of materials with intrinsic antibacterial properties is a promising field of research. We combined chitosan (CS), with antibacterial properties, with polyhedral oligomeric silsesquioxanes (POSS), a biocompatible polymer with physico-chemical, mechanical, and rheological properties, creating a hydrogel using cross-linking agent genipin. The antibacterial properties of CS and CS-POSS hydrogels were investigated against nosocomial Gram-positive and Gram-negative bacteria both in terms of membrane damage and surface charge variations, and finally, the anti-biofilm property was studied through confocal microscopy. Both materials showed a good antibacterial capacity against all analyzed strains, both in suspension, with % decreases between 36.36 and 73.58 for CS and 29.86 and 66.04 for CS-POSS, and in plates with % decreases between 55.29 and 78.32 and 17.00 and 53.99 for CS and CS-POSS, respectively. The treated strains compared to the baseline condition showed an important membrane damage, which also determined a variation of surface charges, and finally, for both hydrogels, a remarkable anti-biofilm property was highlighted. Our findings showed a possible future use of these biocompatible materials in the manufacture of medical and surgical devices with intrinsic antibacterial and anti-biofilm properties.

## 1. Introduction

Healthcare-associated infections (HAIs) are one of the most relevant public health problems worldwide, in terms of both morbidity and mortality, and with a high impact on patient’s safety and healthcare expenditure [1]. In Italy, according to the National Higher Health Institute, 450,000–700,000 HAIs occur each year [2]. Microorganisms responsible of HAIs are often multidrug-resistant (MDRO) or pandrug-resistant (PDRO) [3,4,5] and are grouped under the acronym of “ESCAPE” microorganisms that stands for *Enterococcus faecium*, *Staphylococcus aureus*, *Clostridium difficile*, *Acinetobacter baumannii*, *Pseudomonas aeruginosa*, and *Enterobacteriaceae*. These bacteria colonize hospital environments and surfaces, cross-contaminating medical devices such as intravascular and urinary catheters, prosthesis, etc., being responsible for different types of infections that are hard to manage and fight [6,7]. For these reasons, the scientific community has focused its attention on innovative strategies.

Particularly, devices-related infections are one of the most serious complications of healthcare [8]. Possible sources of these infections are the hospital environment, including operating rooms, medical and surgical equipment, operating staff clothing, and resident bacteria belonging to the patient’s skin microbiota [7,9]. Although some measures such as the control of sterility in operating rooms [10] and antibiotic prophylaxis [11] are able to reduce the incidence of these infections, these strategies cannot completely eradicate the risk of infection. Furthermore, some conditions can increase the clinical severity, including obesity, poor nutritional status, and comorbidities such as rheumatoid arthritis and diabetes mellitus [12,13]. Moreover, an important feature about devices-related infections is that causative strains, including Gram-positive [e.g., *Enterococcus faecalis*, *Staphylococcus aureus* including methicillin-resistant strains (MRSA), *Staphylococcus epidermidis*, and other coagulase-negative staphylococci (CoNS)] and Gram-negative bacteria (e.g., *Klebsiella pneumonia*, *Pseudomonas aeruginosa*, *Acinetobacter baumannii*, *Escherichia coli*, and *Proteus mirabilis*), are often able to produce biofilm [14,15].

Nanotechnologies, due to the possibility to synthesize various nanosized particles and nanomaterials with intrinsic antimicrobial and antibiofilm properties, are receiving great attention and it could help a lot in the fight against HAIs [16,17]. Nanomaterials are compounds with at least one of their dimensions sized less than 100 nm [18] that could represent excellent antimicrobial agents for the prevention of infectious diseases thanks to their intrinsic antibacterial properties. These nanomaterials include, according to the structure, inorganic, carbon-based, and organic nanoparticles [17]. Organic NPs consist of proteins, carbohydrates, lipids, and polymers [19]. Polymer-based nanoparticles can consist of polymers of synthetic or natural origin. The latter, almost all biocompatible, can be polysaccharide-based or protein-based [20].

Among polysaccharide-based polymers that can be used to make nanocomposites there is chitosan (CS), which is also often used for the synthesis of novel multimodal scaffolds, hydrogels, sponges, and membranes for biomedical use, thanks to its good biocompatibility, biodegradability, ease of chemical modifications, antibacterial properties, and high affinity in vivo [21,22]. Especially hydrogels have gained major attention due to their high-water content, softness, flexibility, and antibacterial activity, which made them excellent candidates towards many biomedical applications [23]. Indeed, in recent times, a big variety of antibacterial hydrogels have been developed, some with intrinsic or self-antibacterial activity, such as peptide-based hydrogels, chitosan (CTS)-derived hydrogels, dextranaldehyde/polyethyleneimine hydrogels, and inorganic composite hydrogels [24,25,26,27,28], and some others loaded with antimicrobial materials as hydrogels comprised inorganic nanoparticles, antibiotic-loaded antibacterial hydrogel, photosensitive antibacterial hydrogel, and hydrogels with synergetic effects [29,30,31,32].

CS is a linear polycationic heteropolysaccharide compound of N-acetyl D-glucosamine and D-glucosamine units, nontoxic, and recognized as safe by the United States Food and Drug Administration (FDA) [33]. Its antimicrobial effect is attributed to its positive surface charges, which allow it to interact with the negatively charged bacterial wall, leading to an alteration of transmembrane transport and cellular homeostasis. Furthermore, binding to bacterial DNA is possible, causing inhibition of DNA replication and cell death [34]. For these reasons, CS could have a wide range of applications among which the use in surgery in order to limit the risk of implant and prosthesis infections [35,36,37]. Although medical implants have changed medicine today and bring undoubted benefits, they, however, increase the risk of infection. Any surgical and medical device is an invasive item, and its insertion is able to trigger an immune reaction due to the presence of a foreign body. This condition causes vulnerability to bacterial attack by different pathogens [38].

Many researchers developed chitosan-based hydrogels that, in addition to show the ability to inhibit the growth of pathogenic bacterial cultures such as *E. coli*, *S. aureus*, *P. aeruginosa*, and *Candida albicans* [39,40,41,42], have also been studied in regenerative medicine as compound favoring organ and tissue regeneration [43,44,45]. Unfortunately, the use of CS-based materials shows some limitations related to low mechanical strength, quick hydrolysis, and burst drug release of this polymer which limit its use as single component [46,47]. In order to overcome these drawbacks, CS-based scaffolds have been reinforced with bioactive ceramic materials such as hydroxyapatite, zirconium oxide, titanium dioxide, bioglass ceramics, or silica nanoparticles [48,49,50,51]. The latter are often favored thanks to their mechanical properties that allow an improvement from the point of view of structure, bioactivity, and osteoregeneration [52]. Among nanomaterials of silicon origin, used for this purpose, there are polyhedral oligomeric silsesquioxanes (POSS), that are silicon/oxygen cage structures with (RSiO _3/2_)_8_ repeated units and size range of 1–3 nm. These compounds are characterized by a hybrid chemical composition, intermediate between that of inorganic materials (SiO2) and organic silicone polymers (R 2SiO) [53]. This peculiar structure makes POSS inert, thermally stable, and easily modifiable. Moreover, previous in vitro studies highlighted the high biocompatibility of CS-POSS [35,47]. Thus, POSS, thanks to their good biocompatibility and interesting physico-chemical properties capable of enhancing the mechanical and rheological properties of biopolymers, have shown to be excellent nanofillers for a wide range of biomedical applications for the development of biomedical devices, tissue engineering scaffolds, drug delivery systems, and biosensors [47].

The purpose of this study was to test the potential bactericidal/bacteriostatic effect of CS and CS-POSS hydrogels on hospital bacterial strains isolated by cases of HAIs to demonstrate how the latter maintains an adequate antimicrobial activity and greater stability that allows its use in making medical devices and, especially, in orthopedics, as bone tissue engineering formulations with intrinsic antimicrobial and anti-biofilm properties.

## 2. Materials and Methods

### 2.1. Synthesis of CS-POSS Hybrids

CS-POSS hybrids have been synthesized through Michael type addition reaction [35]. Briefly, CS powder (200 mg) (medium molecular weight and deacetylation degree of 75–85%, Sigma Aldrich, St. Louis, MO, USA) was dissolved in a solution of 2% aqueous acetic acid (10mL) for 30 min at 45 °C and then treated with acryloxypropylheptaisobutyl-POSS (MA0701, C34H72O14Si8, MW: 929.61 g/mol, Hybrid Plastics, Hattiesburg, MS, USA), 200 mg (1 equiv, 0.21 mmol). This mixture was left under magnetic stirring, at reflux (50 °C) for 12 h. The so-obtained CS-POSS was treated with a saturated solution of NaHCO_3_ until neutral pH and then purified through dialysis bags (MW: 12,000 Da) for two days. The purified sample was lyophilized by freeze-drying at −80 °C for 72 h and then used for the subsequent characterizations. As reported, the effective conjugation of POSS with the polymer was confirmed by FTIR spectroscopy and TGA analysis performed under inert atmosphere [35]. The percentages of free amino groups in both CS and CS-POSS hybrid were evaluated by UV–vis absorption spectra, after reaction with ninhydrin measuring the absorbance of the solutions at 570 nm [54] and were found to be of 82% and 45% for CS and CS-POSS hybrid, respectively.

### 2.2. Synthesis of CS and CS-POSS Hybrid Hydrogels

CS powder (200 mg) or CS-POSS (200 mg) were dispersed in a 2% aqueous acetic acid solution for 30 min, at 45 °C. Then, 20 mg (0.1 mmol) of the cross-linking agent genipin (purity > 98%, Carbosynth, St. Gallen, Switzerland) was slowly added to the mixture until the formation of a 3D gel, thanks to the bond of two amino groups between the neighboring chains of the CS polymer. The so-formed hydrogels have been rinsed with deionized water and then stored at 15 °C in a hermetic sealed pan with a constant relative humidity. The water content was evaluated by drying the hydrogels in a beaker for 24 h at 37 °C and at a vacuum drying pressure of 65 mbar until constant weight and was found to be equal to 94 wt% and 78 wt% for CS and CS-POSS hydrogels, respectively. As reported [35], rheological characterizations, namely the frequency response of G0 and the complex viscosity η*, monitored 30 min after the start of crosslinking, were performed for CS and CS-POSS hydrogels. The complex viscosity and the elastic modulus were evaluated as a function of frequency, in the range of 0.01–200 rad/s. From these analyses emerged that the G0 value of CS (at 0.1 rad/s) was of 118,183 Pa, with a viscosity value of 1,197,866 Pa*s, while for the CS-POSS sample, these values were, respectively, reduced to 3684 Pa and 50,183 Pa*s. These results are in agreement with the data obtained from the evaluation of the free amino groups by UV-vis since the decrease of these groups leads to a progressive reduction of the reticulation degree with a consequent decrease in stiffness (reduction in the G0 modulus) and in structural complexity (reduction in viscosity).

### 2.3. Bacterial Strains

Bacterial strains used in this research were detected on samples from patients affected by HAIs admitted at the University Hospital “G. Martino” of Messina, Italy. Specifically, 44 strains were used, of which 38 were Gram-positive [methicillin-susceptible *Staphylococcus aureus* (MSSA), methicillin-resistant *Staphylococcus aureus* (MRSA), methicillin-susceptible *Staphylococcus epidermidis* (MSSE), methicillin-resistant *Staphylococcus epidermidis* (MRSE), vancomycin-susceptible *Enterococcus faecium* (VSE), and vancomycin-resistant *Enterococcus faecium* (VRE)] and 6 were Gram-negative (non-MDR and MDR *P. aeruginosa* (PSEAER)) bacteria. The used bacteria were isolated by clinical specimens using common growth media specific for different strains. In particular, Mannitol Salt Agar (MSA), Bile Esculine Agar, and MacConkey Agar in aerobic conditions and incubated at 37 °C for 24 h were used for staphylococci, enterococci, and *P. aeruginosa*, respectively. After detection, the strains were identified, and the antimicrobial susceptibility was evaluated using the automatized system VITEK^®^ 2 COMPACT (bioMérieux Clinical Diagnostics). After that, strains were stored at −20 °C in Luria-Bertani (LB) Miller formulation Broth with 15% glycerol. For their use, strains were unfrozen and plated on the same agar plates used for their detection. The plates were, then, incubated at 37 °C for 24 h. From these subcultures on solid plates, a suspension in Mueller-Hinton Broth (MHB) (BD DIFCO™, Franklin Lakes, NJ, USA) with an OD of 0.5 was obtained.

### 2.4. Antimicrobial Activity of CS and CS-POSS

The antimicrobial activity of the investigated materials was tested both in liquid phase, by dispersing the materials in the bacterial suspension, and in solid phase, by inserting the CS or CS-POSS hydrogel with dimensions of about 2 cm^2^ (800 µg·mL^−1^), on the plate, and left to solidify. In particular, the treatment in the liquid phase was carried out by adding 200 µL of CS or CS-POSS (4 mg·mL^−1^) to 1 mL of bacterial suspension with OD 0.5 on MHB. The suspension grew for 24 h at 37 °C under stirring to ensure a good dispersion of the materials. After 24 h, the OD of each treated and untreated suspension was measured. With regard to the solid phase treatment, 10 μL of bacterial suspensions with OD 0.5 were plated on Muller-Hinton agar medium. The plates were, then, incubated at 37 °C for 24 h. After the incubation, the growth on the plate without and with hydrogel was evaluated and the ODs from the suspensions derived from the two plate sections were measured.

All OD measures were performed in triplicate and the average values were reported with standard deviations.

### 2.5. PI Assay to Monitor Microbial Membrane Permeability

The evaluation of membrane permeability was performed using propidium iodide (PI) fluorochrome according to the literature [55]. Specifically, bacteria were grown for 24 h at 37 °C in MHB then harvested, washed, and resuspended in a buffer solution containing 5 mM glucose and 5 mM HEPES at pH 7.2 to an OD 600 nm value of ~0.25. From this bacterial suspension, 150 μL was added to wells of a 96-well plate and 10 μL of PI solution (50 μM) was added and preincubated for 10 min. Following preincubation, florescence was measured for the next 8 min with a time interval of 2 min using a microplate reader (535 nm excitation, 617 nm emission, Tecan, Switzerland). After this, 30 μL of CS or CS-POSS (800 μg·mL^−1^) were added, and the florescence intensity was monitored after 10, 20, and 30 min. The assay was performed in three replicates, and the average values ± SD were reported.

### 2.6. Bacterial Cell-Surface Properties

To evaluate the effects of CS or CS-POSS on bacterial cell surface charges, we used a modified version of the hydrocarbons test (MATH) as described by Zanane et al. [56]. In detail, we used the solvents ethyl acetate (as donating electron) and chloroform (as accepting electron) to evaluate any changes of charges on the cellular surface. Briefly, bacterial cells grown overnight in MHB at 37 °C, without and with CS and CS-POSS (800 μg·mL^−1^), were harvested by centrifugation (6000 rpm for 10 min), washed twice with PBS, and resuspended in such a volume of PBS to obtain a bacterial suspension with an OD400 nm between 0.5 and 0.7 (A0). Aliquots (3 mL) of each treated and not-treated bacterial suspension were added to each tube containing 0.4 mL of the ethyl acetate, a strong basic solvent, and chloroform, an acidic solvent which exhibits negligible basic character. After vigorous agitation by vortex, phases were allowed to separate for 10 min at 30 °C and the OD400 nm of the aqueous phase was measured (A1).

The percentage of affinity to each solvent was calculated as follows:

$$\%\;\mathrm{Affinity}=\frac{A0-\;A1}{A0}\times 100$$

### 2.7. Confocal Microscopic Observation of Biofilm

The inhibitory capacity of CS and CS-POSS against bacterial biofilm was investigated by laser scanning confocal microscopy (CLSM). As described by Spanò et al. [57], aliquots of overnight cultures in MHB (adjusted to OD600 = 0.5) were distributed in chamber slides (Nunc Inc., Naperville, IL, USA), previously coated with CS and CS-POSS, to the CLSM observations. After incubation at 37 °C for 24 h, the suspended bacteria were eliminated and the remaining cells adherent to the slide, after washing with phosphate buffered saline (PBS), were heat fixed and finally stained with 20 μg·mL^−1^ Propidium Iodide (PI) (Sigma), an intercalating of nucleic acids that has the fluorescence excitation maximum and the emission maximum equal to 535 nm and 617 nm, respectively. The slides were incubated in the dark at 30 °C for 5 min to allow the fluorescent labeling of the bacteria. The observations were performed by CLSM using a TCS SP2 microscope (Leica Microsystems Heidelberg, Mannheim, Germany), equipped with an Ar/Kr laser and coupled to a microscope (Leica DMIRB). *S. aureus* was considered a representative target bacterium.

### 2.8. Statistical Analyses

Statistical analyses were performed using Prism 4.0 software (GraphPad, San Diego, CA, USA). Stratified data were statistically analyzed using one-way ANOVA and *t*-tests. Pearson’s correlation test was used to determine any correlations between the studied variables. Significance was assessed at the *p* < 0.05 level.

## 3. Results

### 3.1. Antibiotic Susceptibility of the Tested Strains

All the susceptibility/resistance patterns of the used bacterial strains to the antibiotics are shown in Table 1.

The table shows a diversified antimicrobial pattern. As to be expected, resistant strains showed a higher average percentage of total resistance compared to the sensitive strains. Specifically, VRE resulted by far the most resistant strains among the resistant ones and, in general, enterococci were most resistant compared to the other strains.

### 3.2. Antibacterial Properties of CS and CS-POSS

**Figure 1:**
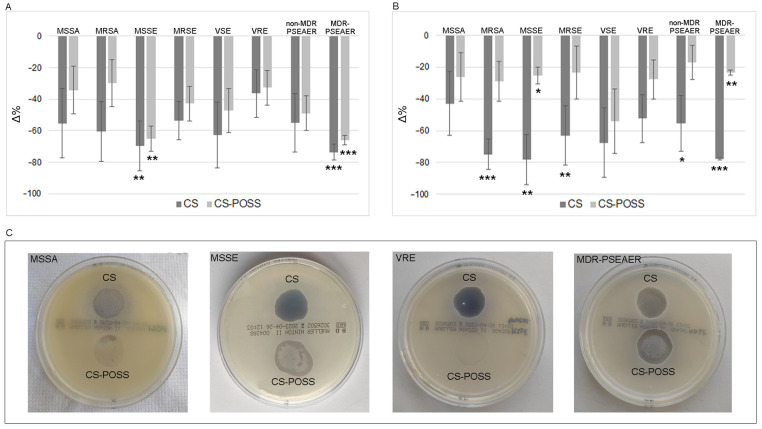
Decreased bacterial growth in the CS and CS-POSS, compared to baseline conditions, in the MHB suspension (**A**) and Muller–Hinton agar medium plates (**B**). Bacterial growth in plates with CS and CS-POSS hydrogels (**C**). *, **, and *** *p* < 0.05, 0.01 and 0.001, respectively.

The antimicrobial activity of the tested materials was highlighted both in suspension and on plates for all the tested strains. As shown in Figure 1A, treatment with CS and CS-POSS in MHB showed an important decrease of bacterial growth, albeit in the presence of a certain variability among the strains. In particular, the presence of CS caused a decrease of −55.26% and −60.63% for MSSA and MRSA, −69.56% (*p* < 0.01) and −53.60% for MSSE and MRSE, −62.75% and −36.36% for VSE and VRE, and finally, −54.90% and −73.58% (*p* < 0.0001) in non-MDR-*P. aeruginosa* and MDR-*P. aeruginosa*. The decrease, albeit more contained, was also highlighted by the CS-POSS treatment, and was equal to −34.21% and −29.86% for MSSA and MRSA, −65.21% (*p* < 0.01) and −42.74% for MSSE and MRSE, −47.06% and −32.73% for VRE and VSE, and finally, −49.02% and −66.04% (*p* < 0.0001) for non-MDR and MDR *P. aeruginosa*.

Exposure to the hydrogel confirmed the antimicrobial activity of the tested materials. In particular, despite the high inter-strain variability, there was a remarkable decrease in bacterial growth for CS, with values always above 40% (Figure 1B). The highest decreases were highlighted for MRSA (−74.99%; *p* < 0.0001), MSSE (−78.32%; *p* < 0.01), MRSE (−62.96%; *p* < 0.01), non MDR-*P. aeruginosa* (−55.29%; *p* < 0.05), and MDR *P. aeruginosa* (−77.64%; *p* < 0.0001). The decreases obtained after exposure to CS-POSS hydrogel were rather homogeneous for all the tested strains, with values between −17.00 and −29.00%, with the exception of the VSE which was more sensible to the treatment showing a decrease of −53.99%. Figure 1C shows representative plates, with CS and CS-POSS, of the tested strain, where a more or less marked reduction in bacterial growth on the surface of the hydrogel compared to the rest of the plate is evident. No significant difference in antibacterial properties of tested materials was found between antibiotic susceptible (−62.13% for CS and −32.71% for CS-POSS) and resistant (−61.49% for CS and −25.37% for CS-POSS) strains.

### 3.3. Antibacterial Mechanism

**Figure 2:**
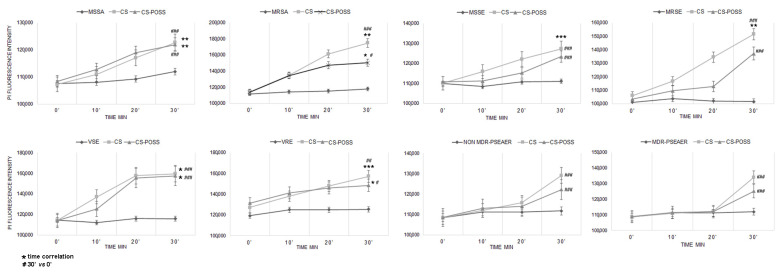
Propidium iodide fluorescence values in basal condition and after 10′, 20′, and 30′ of CS and CS-POSS treatment (* *p* < 0.05, ** *p* < 0.01, *** *p* <0.001; ^#^ *p* < 0.05, ^##^ *p* < 0.01, ^###^ *p* <0.001).

The DNA-binding PI fluorochrome remains silent in the presence of microbes with intact membranes but exhibits strong florescence if the membrane is disrupted, allowing it to intercalate with DNA. As shown in Figure 2, CS and CS-POSS treatment caused a florescence increase of bacterial strains, suggesting that the antimicrobial activity of these materials is attributable to bacterial wall/membrane damage.

The obtained results showed a membrane damage that occurred rapidly in all the tested strains, but with different membrane permeability among microbial species, likely due to the distinct cell surface structures and compositions.

In particular, fluorescence significantly increased with exposure time in MSSA, MRSA, VSE, and VRE for both the materials (*p* < 0.05). In MSSE and MRSE, there was a significant increase in fluorescence after CS treatment, while for the strain treated with CS-POSS, there was a smaller and non-significant increase. Concerning non MDR- and MDR *P. aeruginosa*, there was a delay in the entry of the fluorochrome which was evident only after 30′ of exposure, probably attributable to the characteristic composition of the bacterium, equipped with a dense mucous layer that can make it difficult for exogenous substances to pass through. Even in the absence of a correlation with the exposure time, there was a significant fluorescence increase at time 30′ compared to time 0 for all the tested strains.

**Figure 3:**
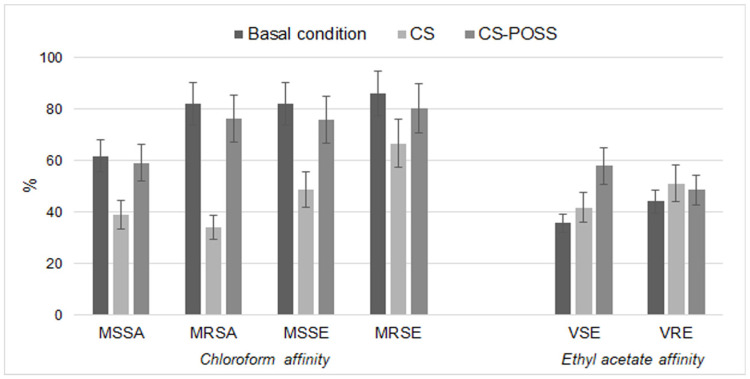
Changes of charges on the bacterial cell surface evaluated by affinity to solvents ethyl acetate (donating electron) and chloroform (accepting electron), after CS or CS-POSS treated.

The affinity to chloroform and ethyl acetate was studied to evaluate the effects of CS and CS-POSS on surface bacterial charges. The experiments showed a reduced chloroform affinity that was marked for CS and almost superimposable on the control for CS-POSS, indicating a variation of surface charges, only for staphylococci. As shown in Figure 3, the treatments with the materials under study determined a lower affinity towards chloroform in staphylococci, in comparison to basal conditions. In particular, for MSSA, an affinity of 39.00% and 59.11% after CS and CS-POSS treatment was found, respectively. For MRSA, CS treatment reduced the affinity to chloroform to 34.15%, while CS-POSS treatment reduced the affinity to 76.40%. The MSSE and MRSE chloroform affinity was reduced to 48.97% and 66.67% for CS treatment and 76.13% and 80.28% in CS-POSS treatment. Regarding VSE and VRE, the results showed that treatment with CS and CS-POSS resulted in a higher affinity for ethyl acetate, with values equal to 41.94% and 58.00% for VSE and 51.24% and 48.65% for VRE (Figure 3). No changes in surface bacterial strain were recorded for non-MDR-PSEAER and MDR-PSEAER compared to the control strain.

### 3.4. Inhibitory Properties of CS and CS-POSS towards Biofilm Formation

**Figure 4:**
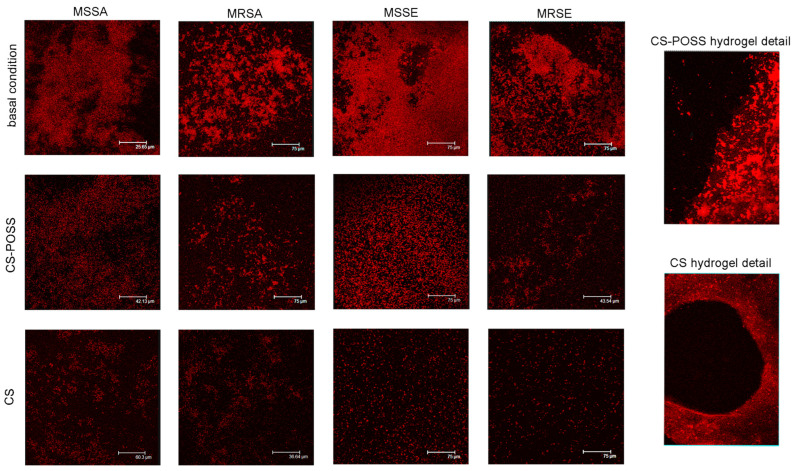
Confocal laser images of biofilm formed by *S. aureus* in basal condition and with CS and CS-POSS hydrogels.

The inhibitory property of CS and CS-POSS towards bacterial biofilm formation was tested on *S. aureus* as target bacterium. Compared to basal condition, whose biofilm showed compact structure with well-clustered internal bacterial cells, the CLSM images showed how CS-POSS and especially CS treatment were able to destroy the dense biofilm structure in MSSA, MRSA, MSSE, and MRSE (Figure 4). The figure also shows in detail the border area between the hydrogel and the free surface, highlighting a remarkable reduction or complete absence of the bacterium above the CS and CS-POSS hydrogels, respectively.

## 4. Discussion

HAIs are nowadays a crucial issue for the global public health involving a very high number of patients in different settings with concerns in terms not only of morbidity and mortality but also of healthcare assistance and sanitary costs [58]. For these reasons, many efforts are to date addressed to find new and innovative solutions to fight them. In this scenario, next to the classic actions consisting of good surgical, assistance and hygiene practices, new technologies, and manufacturing processes, the discovery of new materials, the development of medical devices with modified surface, and the use of different polymers have attracted remarkable attention in recent years by the scientific community with the purpose to produce devices with superior properties and morphology [59,60].

In the wide group of HAIs, infections caused by contaminated medical and surgical devices are very important because of their capacity to nullify the benefits of healthcare due to the fact that an infected device must be necessarily removed and an antibiotic treatment carried out. For the infection of prosthesis, for example, the patient often must be subjected to a new surgery with all the possible side effects of a new anesthetic treatment. Moreover, the infection can become systemic putting the patient at risk of sepsis and septic shock especially considering co-morbidities affecting very often this kind of patient [61].

Our study had the purpose of evaluating the in vitro antibacterial activity of CS and CS-POSS, materials that have a good biocompatibility and poor cytotoxicity and that have already been studied for biomedical use [59]. Moreover, the antimicrobial properties of CS have been highlighted by several studies [62,63,64]. However, the use of this compound as potential biomaterial for medical devices is still a poorly studied issue. In the light of a future use of CS in this context, the addition of POSS would be essential in order to give support to CS and increase its mechanical resistance. POSS belongs to the family of organic-inorganic hybrid materials that has attracted much attention due to their thermal, mechanical, and flame retardation properties [65,66]. POSS structure contains a stable Si-O inorganic core, so it is an ideal compound suitable for the production of polymeric nanocomposite with improved physico-chemical properties. Moreover, its substituents can be modified adding several chemical groups with different polarities and functionality able to enhance its structural, mechanical, thermal, biocompatible, permeability, and oxidative properties and stabilize the flame retardance capacity [67,68,69,70,71].

In our evaluation, these compounds showed a very important and interesting antibacterial activity both as liquid suspension and in solid phase (hydrogel). We decided to use strains detected by cases of HAIs focusing our attention especially on Gram-positive bacteria and in staphylococci in particular. We further decided to choose both antibiotic sensitive and resistance strains in order to evaluate if an antibiotic-resistance could affect the CS and CS-POSS sensitivity. Actually, no significant differences were detected between the two groups, suggesting that resistance to antibiotics does not affect resistance to the two studied materials. This finding is a very important result, considering that very often HAIs are caused by hospital strains resistant to many drugs used for their treatment.

Actually, in the antibacterial activity, CS was always more effective than CS-POSS, especially in solid phase. This is easily attributable to the different percentage of free amino groups present on the polymer before and after the conjugation reaction (which resulted to be 82% and 45% for CS and CS-POSS, respectively); therefore, the ability of the CS to interact with the bacterial surface is reduced, which is known to be the main route of antibacterial action [64]. However, despite this result, the reduction of bacterial growth obtained with the use of these nanocomposites is without any doubt a remarkable finding that could be of great help in counteracting the onset of HAIs. The antimicrobial activity of CS-POSS in suspension showed significant percentage decreases in exposed strains, in line with what Li et al. [55] found for guanidinium-perfunctionalized POSS against *S. aureus*. This activity was more contained in plate, probably solidification of CS-POSS is a further weak point reducing the interaction with bacterial cells, and it is superimposable as regards *S. aureus* with data present in the literature relating to other highly promising hydrogels showing, by plate counting approach, an antibacterial effect equal to 23% [72] in melanin-reinforced biopolymer hydrogel, and of 28% in hydrogel (QOP) composed of polysaccharide matrix (quaternized chitosan and oxidized β-glucan) and polydopamine nanoparticles [73].

Further experiments carried out using PI confirmed that membrane damage has surely one of the most important antibacterial mechanisms showed by the materials under study. Even in this case, CS-POSS showed a lower damage activity in total agreement with the performed liquid and plate tests. Anyway, for many treated strains, there was a significant difference with the untreated control strain showing a still valid activity. The loss of cellular integrity found in our study is in line with that reported by Xiang et al. [72] that show in bacteria exposed to melanin-reinforced biopolymer hydrogel, crumpled cell walls, and cell membranes, because the positively charged hydrogel can alter the bacterial membrane potential and curb their metabolism by electrostatic adsorption. In addition, the experiments carried out using chloroform and ethyl acetate showed a change in surface charges in staphylococci and enterococci. Specifically, we found a lower affinity of treated strains to chloroform, as further confirmation of the binding of CS to the bacterial surface.

Very interestingly, both CS and CS-POSS were able not only to reduce the bacterial growth but also to effectively act against the biofilm formation, which is a crucial step in the colonization of medical devices shown by many important pathogens causing HAIs [8,74]. Especially in device-induced infection, biofilm formation is extremely important and to have some materials able to counteract this step could make the difference in the fight against this kind of infections [15]. Confocal images clearly showed the anti-biofilm activity of CS and CS-POSS especially for some strains such as MRSA and MRSE. When a device is colonized, the biofilm formation causes an increased resistance to antibiotic treatments [75], as well as tolerance to disinfectants and resistance to phagocytosis by the immune system. In these cases, the infection is not manageable using conventional techniques, and the treatment includes two stages: (1) the device must be removed and the infection treated, and (2) a new device must be implanted [14].

The use of nanomaterials with intrinsic antimicrobial activity will allow to overcome one of the most worrying concerns about HAIs treatment: the difficulty to discover and/or artificially create new antibiotic molecules able to effectively kill bacteria more and more drug-resistant and capable of persisting in the hospital environment creating risk situation of device cross-contamination. Our study shows that biopolymers such as CS and the derivative CS-POSS exhibit antibacterial and anti-biofilm properties on clinical bacterial strains. This result, in addition to other characteristics already demonstrated by us, such as chemical, physical, and rheological properties, drug release, and biocompatibility, makes CS-POSS hydrogel a promising material in the fabrication of medical-surgical devices.

## 5. Conclusions

Some nanomaterials and nanocomposites have very interesting antimicrobial properties and for this reason they represent potentially promising tools in the fight against HAIs. Their use in the manufacturing process of medical and surgical devices could help the scientific community to contain this worrying and pervasive phenomenon. Particularly, CS-POSS hydrogels have shown important antibacterial and anti-biofilm capacity and these activities, combined with its biocompatibility and versatility, make it a particularly promising material for this purpose. The development of these compounds and their use in the manufacturing of devices and surfaces with intrinsic antimicrobial and anti-biofilm properties could be of great help in reducing the burden of HAIs, even in the light of overcoming the real problem of the lack of new antibiotics, effective in treating infections caused by multidrug-resistant bacteria. Moreover, this possible future scenario, i.e., avoiding the burden of HAIs, could be useful to reduce the huge amounts of antibiotics now used to treat and manage this kind of infection. This possibility would surely have great positive aspects for patients, reducing on the one hand morbidity and mortality from HAIs and on the other hand avoiding antibiotic treatments too often long and not free from side effects, especially on fragile patients. Future research in contrast to HAIs will be more and more focused on these new materials, with great positive achievements for healthcare and global public health.

## Figures and Tables

**Table 1:** Antimicrobial resistance patterns of the used bacterial strains.

	MSSA (7)	MRSA (6)	MSSE (4)	MRSE (9)	VSE (5)	VRE (7)	Non-MDR PSEAER (3)	MDR PSEAER (3)
**Amikacin**	--	--	--	--	--	--	0%	0%
**Amoxicillin Clavulanate**	--	--	--	--	50%	100%	--	--
**Ampicillin**	--	--	--	--	50%	100%	--	--
**Ampicillin/Sulbactam**	--	--	--	--	50%	100%	--	--
**Benzylpenicillin**	71.4%	85.7%	--	--	--	--	--	--
**Cefepime**	--	--	--	--	--	--	0%	50%
**Cefoxitin**	--	100%	--	--	--	--	--	--
**Ceftaroline**	0%	0%	--	--	--	--	--	--
**Ceftazidime**	--	--	--	--	--	--	0%	50%
**Ceftazidime/Avibactam**	--	--	--	--	--	--	0%	0%
**Ciprofloxacin**	--	--	--	--	50%	100%	33.3%	0%
**Clindamycin**	28.6%	71.4%	0%	55.6%	--	--	--	--
**Colistin**	--	--	--	--	--	--	0%	0%
**Co-trimoxazole**	0%	0%	0%	0%	--	--	--	--
**Daptomycin**	0%	0%	0%	0%	--	--	--	--
**Erythromycin**	28.6%	71.4%	50%	77.8%	--	--	--	--
**Fosfomycin**	--	--	--	--	--	--	33.3%	--
**Fusidic acid**	14.3%	0%	25%	66.7%	--	--	--	--
**Gentamicin**	0%	0%	50%	66.7%	0%	71.4%	0%	0%
**Imipenem**	--	--	--	--	50%	100%	0%	100%
**Kanamycin**	--	--	--	--	50%	71.4%	--	--
**Levofloxacin**	0%	28.6%	0%	88.9%	50%	100%	--	--
**Linezolid**	0%	0%	0%	11.1%	0%	0%	--	--
**Meropenem**	--	--	--	--	--	--	0%	0%
**Mupirocin**	0%	0%	--	--	--	--	--	--
**Oxacillin**	0%	100%	0%	100%	--	--	--	--
**Piperacillin/Tazobactam**	--	--	--	--	--	--	0%	50%
**Quinupristin/Dalfopristin**	--	--	--	--	25%	0%	--	--
**Rifampicin**	0%	0%	0%	11.1%	--	--	--	--
**Streptomycin**	--	--	--	--	50%	71.4%	--	--
**Teicoplanin**	0%	0%	0%	44.4%	0%	100%	--	--
**Tetracycline**	0%	14.3%	75%	22.2%	--	--	--	--
**Tigecycline**	0%	0%	0%	0%	0%	14.3%	--	--
**Tobramycin**	--	--	--	--	--	--	0%	0%
**Vancomycin**	0%	0%	0%	0%	0%	100%	--	--
**Average Value**	**8.4%**	**26.2%**	**14.3%**	**38.9%**	**30.4%**	**73.5%**	**5.5%**	**22.7%**

## Data Availability

Not applicable.
